# Looking at the Process: Examining Creative and Artistic Thinking in Fashion Designers on a Reality Television Show

**DOI:** 10.3389/fpsyg.2018.02008

**Published:** 2018-10-23

**Authors:** Jillian Hogan, Kara Murdock, Morgan Hamill, Anastasia Lanzara, Ellen Winner

**Affiliations:** ^1^Department of Psychology, Boston College, Chestnut Hill, MA, United States; ^2^Department of Psychology, Northeastern University, Boston, MA, United States; ^3^Department of Psychology, University of Bath, Bath, United Kingdom

**Keywords:** creative thinking, habits of mind, fashion design, reality television, Project Runway

## Abstract

We examine creativity from a qualitative process rather than a quantitative product perspective. Our focus is on “habits of mind” (thinking dispositions) used during the creative process, and the categories we used were those of the eight Studio Habits of Mind observed in visual arts classrooms (Hetland et al., 2007, 2013). Our source of data was footage from a popular reality television show, *Project Runway*, in which nascent fashion designers are given garment design challenges. An entire season of the show (14 episodes) was transcribed and coded for the presence of eight Studio Habits of Mind. We found abundant evidence of all eight of these thinking dispositions in all portions of the show. We argue that the creative thinking occurring during fashion design bears strong resemblances to that which occurs in the art studio-classroom. Qualitatively created frameworks, like those of the Studio Habits of Mind, can be used to inform our understanding of creative behavior in various disciplines.

## Introduction

The current emphasis in creativity research is on what Glãveanu (2014) calls the “quantification of creativity” (p. 22) – the overwhelming proportion of creativity assessment is measured through quantitative psychometrics. But creativity is a complex, multi-sensory, and situation-dependent phenomenon, not easily captured in a numerical value. Here, we argue that creative behavior can and should be examined through a rigorous and systematic qualitative lens during the act of authentic creation. In short, we should be analyzing *processes* of creative thinking and activity, alongside ongoing work in assessing created products. Our view is shaped by conceptions developed by researchers in the field of education, and specifically in the field of primary and secondary school visual art education.

### Concepts of Process and Product

The dichotomy between process and product is a familiar one in the field of education (Bruner, 1960; Lachman, 1997; Runco, 2003), and particularly in visual art education (Sullivan, 2001; Gude, 2010; McLennan, 2010). Educators must balance teaching and assessing concrete technical skills, which often lead to polished products, versus teaching and assessing creative thinking potentials, which are often exhibited through exploratory, messy processes, as discussed by Sawyer (2017).

We focus here on the discipline of arts education and argue for a process-based rather than product-based approach to examining creative thinking in the arts. In a product-based view, the artwork is paramount in assessing a student. These works may be assessed on various dimensions – e.g., technique, expression, realism, composition, etc. While this lens offers some information about the student’s skills and interests, arts educators have countered that a process-based view is one that provides an alternative lens that is informative in ways that final products cannot capture.

In a process-based view, the final “product” is the artistic mind of the student (Hetland et al., 2007, 2013). The authentic behaviors, motivations, and awareness of various thinking dispositions that are useful in the domain are only accessible through close observation of students at work, or through evidence of their reflection on their making process (through conversation, critique, and written artist statements.) In short, a process-based view is not one that depends solely on any particular tangible artifact that can be ranked, counted, or numerically measured. Rather, it is one that requires attention to the ways a student thinks and how those thoughts form habits of cognition and behavior. These observations and reflections form evidence of thinking in the act of making (or the student’s artistic mind).

Like art educators, psychologists have also categorized creative thinking in terms of both product and process. Additional categories include personality and press [or environment], constituting the 4 Ps (Fishkin and Johnson, 1998; Barbot et al., 2011; Said-Metwaly et al., 2017). But in psychology, even those approaches to creativity assessment that are “process” based are essentially dependent upon what educators would think of as products. The most processual approaches are those that aim to measure the cognitive aspects that can eventually lead to creative behavior – most namely, divergent thinking. Process-based approaches include tests like the Remote Associations Test (Mednick and Mednick, 1967), the Structure of the Intellect divergent production tests (Guilford, 1967), the Wallach-Kogan Creativity Tests (Wallach and Kogan, 1965), and the Torrance Tests of Creative Thinking (Torrance et al., 1966). These measures of creativity examine characteristics such as number of ideas, uniqueness, or level of detail in generated drawings, writings, and verbalizations. It is argued that the divergent thinking captured in these tasks is one aspect of the process that can lead to the creation of creative products. However, the quantitative paradigm of psychology’s process approach is very different from art education’s depictions of process, which focuses more heavily on qualitative data collection, analysis, synthesis, and assessment of individual growth. When we discuss process here, we refer to understandings from the discipline of art education, which we believe can be applied to creativity research at large as a complement to existing approaches.

### Current Creativity Approaches

There are no perfect measurements of creativity. However, when process and product, qualitative and quantitative, or subjective and objective measures are combined, each approach complements the other. This is especially true for a construct like creativity, which is complex (Cropley, 2000; Barbot et al., 2011), ill-defined (Plucker et al., 2004), and changes with historical and/or discipline-based lenses (Hennessey and Amabile, 2010; Barbot et al., 2011). Given the relatively slow progress in the area of creativity assessment in comparison to other areas (Plucker and Makel, 2010), we support the view that varied approaches to assessment allow for methods to be more widely tested and help advance the field (Silvia et al., 2012).

Within the 4 Ps of approaches in psychology (Fishkin and Johnson, 1998; Barbot et al., 2011; Said-Metwaly et al., 2017), each category has benefits and drawbacks.

#### Psychology’s Process Approach

While process-focused psychological approaches described above are generally accepted as reliable (Cropley, 2000), their validity is debated (for discussions of validity, see Hocevar and Bachelor, 1989; Cropley, 2000; Simonton, 2003; Clapham, 2004; Said-Metwaly et al., 2017). This issue is put plainly by Glãveanu (2014, p. 16), who writes: “How is [the] experiential and ontological richness of creativity as a phenomenon ever contained in tasks like ‘please generate as many uses as possible for a brick’?” As Said-Metwaly et al. (2017) note, process-focused approaches (and all other currently accepted approaches) suffer from a limited scope in what they measure; therefore the use of only one approach will fail to capture the complexity of creative behavior.

#### Psychology’s Product Approach

Product-focused approaches are those in which products of a task are assessed using the Consensual Assessment Technique (CAT; Amabile, 1982). In CAT, a social psychological perspective is taken – a team of judges who are experts within the domain independently determine whether and to what degree a product is “creative.” This approach is generally highly reliable and valid (Baer et al., 2004; Kaufman et al., 2007; Said-Metwaly et al., 2017). However, this approach can be time-consuming and expensive, requiring skilled judges. Teams of non-experts do not produce consistent or reliable ratings (Kaufman et al., 2008), and thus findings from CAT depend upon the opinion of experts in the field, which may or may not align with perceptions of the general public or experts from other domains. Because this is a subjective approach, results are limited to the historical and socio-cultural contexts at the time of judging (Amabile, 1982).

#### Psychology’s Personality Approach

Personality-focused approaches constitute the third P. These consist primarily of self-report questionnaires about qualities associated with creative people (i.e., attraction to complexity, high energy, behavioral flexibility, non-conformity, self-esteem, self-acceptance, risk taking, perseverance, introversion, the inclination to connect abstract ideas, and tolerance for ambiguity [Barron and Harrington, 1981; Feist, 1998; Selby et al., 2005; Barbot et al., 2011]) or self-reports of creative accomplishments. Examples of these types of measures include the Creative Personality Adjective Checklist (Gough, 1979), the Creative Perception Inventory (Khatena and Morse, 1994), the Creative Achievement Questionnaire (Carson et al., 2005), and the Runco Ideational Behavior Scale (Runco et al., 2001). Personality-focused approaches are usually standardized and objectively scored and are accepted as highly reliable (Gough, 1979; Said-Metwaly et al., 2017). Like all self-reports, however, findings are biased by participants’ views. Some studies have also shown these measures to lack construct validity (Said-Metwaly et al., 2017). These measures are argued to assess stable traits, which means that this approach does not capture the notion that creativity is something that can be developed (Fishkin and Johnson, 1998). Additionally, Silvia et al. (2012) report that many of these measures result in skewed scores and therefore require careful analysis.

#### Psychology’s Press Approach

The press approach focuses on the environmental factors that come into play when creative behavior is enacted. This is the most historically recent approach to examining creativity assessment and relies on research linking aspects of environmental situations to increased or decreased creativity (Hunter et al., 2007; Hennessey and Amabile, 2010). Like the approach we suggest here, much of the research in this area focuses less on a spirit of *assessment* (connoting ranking, sorting, or other categorizations) and more on *examination* (looking for characteristics), though measures have been created that look for how or less creativity-conducive an environment is or is perceived to be (e.g., KEYS: Assessing the Climate for Creativity, Amabile et al., 1996; the Situation Outlook Questionnaire, Isaksen et al., 2001; and the Virtual Team Creative Climate Instrument, Nemiro, 2001). This approach and these instruments call out for more research, particularly because many are dependent upon subjective judgment.

Here, we take a different approach to what has been discussed. We ask, *how do creative people act and think while engaged in creative behavior*? And *can we systematically capture the thinking dispositions of creative people as we observe them at work?* We believe our method falls outside the scope of the 4 Ps, and acknowledge that, like all current approaches to assessing creativity, this method contains both strengths and limitations. We consider these matters in the Section “Discussion.” In arguing that there are observable behaviors that govern creative behavior, we rely on concepts of disciplinary thinking, or habits of mind, which have been developed within the field of education.

### Disciplinary Thinking

Teachers who assess children, both summatively (as on a report card) and formatively (as part of ongoing feedback during classroom conversations or contained in notes written on an essay or exam), face the same challenges that psychologists do when evaluating skills (both in creativity and other areas). What precisely should be assessed? A final, tangible product like an artwork, essay, or problem set? Effort, participation, and attitude? The intention behind the work? Or technical skills, like how well one shades color values, recites times tables, or constructs clear prose? If a combination, in what proportion?

Some address these matters by choosing to teach and assess habits of mind within general education (Costa and Kallick, 2008; Ritchhart et al., 2011; Root-Bernstein and Root-Bernstein, 2013), discipline-specific education (Hetland et al., 2007, 2013 [art]; Cuoco et al., 1996 [math], Çalik and Coll, 2012 [science]; Epstein, 2003; Lunney, 2003 [medicine]) and in creativity education (Lucas and Spencer, 2017). In this way, the thinking process (traditionally viewed simply as a means to an end product) becomes the primary evidence of learning (in other words, the product of education). The *process* and *product* become blurred: evidence of the thinking process is used to determine what and how a student has learned or grown. Additionally, teachers consider a student’s *personality* or proclivities as part of assessments – if students are naturally inclined to explore, to draw realistically, or to reflect thoughtfully on their process, then teachers may push them harder than they push others in the effort to enhance these inclinations, or to use those strengths as leverage for areas of weakness (Hogan et al., 2018, p. 108–133). These are context dependent judgments, similar to *press* approaches. A teacher knows the time spent creating on a hot Friday afternoon in June will likely yield inferior work to that created on a crisp Tuesday morning in October. The life cycle of the school year, the weather, and special events all play into the ways teachers approach the examination of their students and their thinking and growth. Considered this way, teachers seem to use pieces of each of the 4 Ps, but their wholistic approach cannot easily be captured by the use of any one of these. The approach we describe here is a systematic example of some of the pieces of the assessment process that teachers use in the visual art classroom every day (for examples, see Hogan et al., 2018).

Students can be encouraged to develop thinking dispositions that form part of the creative artistic process. Developing disciplinary thinking in education has been emphasized and described by many (e.g., Gardner, 1999; Lévesque, 2008; Rantala, 2012) and focuses on the processes of thinking authentically in a particular discipline (often through inculcating habits of mind or thinking dispositions). For instance, history teachers can strive to teach students to think like historians and to consider how to make arguments from historical data; and science teachers can encourage students to form testable hypotheses as do scientists.

### Studio Thinking

The approach we use here is based on a framework developed by educational and developmental psychologists studying the kind of creative disciplinary thinking developed in studio art classrooms at the high school level (Hetland et al., 2007, 2013). The Studio Thinking framework identified eight habits of mind – broad types of disciplinary thinking – taught in the studio art classroom, as shown in Table 1 (Hetland et al., 2007, 2013). This framework was developed from the ground up: researchers videotaped, transcribed, and thematically coded utterances of five high school art teachers during many class periods. These teachers were also practicing artists, and taught in arts-centered high schools. The Studio Thinking framework has been adopted by visual arts teachers all over the world, at all levels of primary and secondary school education. Teachers use this framework to teach and assess the thinking processes that students use in their artmaking (Hogan et al., 2018).

**Table 1:** Studio Habits of Mind in the visual art classroom (in alphabetical order; Hogan et al., 2018).

Studio Habit	Sub-habit	Definitions
Develop craft	Technique	Learning to use tools, materials, and artistic conventions
	Studio practice	Taking care of tools, materials, works, and workspace
Engage and Persist	n/a	Finding personally meaningful projects and sticking to them
Envision	n/a	Imagining what cannot be seen and a plan to create artwork of these imagined ideas
Express	n/a	Making works that convey personal meaning
Observe	n/a	Looking closely and noticing what might not ordinarily be seen
Reflect	Question and Explain	Talking about work and working processes
	Evaluate	Talking about what works well, what does not, and why, in works by self and others
Stretch and Explore	n/a	Trying new things, making mistakes, and learning from them
Understand Art Worlds	Domain	Learning what artists have made
	Communities	Learning to collaborate and understanding that artists often work in teams


The eight Studio Habits of Mind are forms of disciplinary thinking in the visual arts. Habits of mind, or thinking dispositions, encompass not just the skill to complete a task (*Can* the student do it?), but also the attitudes that interact with those skills (*Will* the student do it? Does the student know *when* and *why* to do it?; Perkins et al., 1993; Hetland et al., 2007, 2013; Hogan et al., 2018). If a person uses a habit of mind, this can best be seen through authentic observation of the person working naturally. Only through making artistic decisions independently can a person’s motivation, awareness, and other attitudes be observed. In many testing situations, and some teacher-centered environments in education, students are not given the opportunity to make decisions or exhibit the attitudinal aspects of a thinking disposition. Instead, they simply follow directions. Through observation of habits of mind, we look not just for discrete skills but also the attitudes that allow those behaviors to be enacted into the practice of creative work. We consider this to be more ecologically valid – if skills, behaviors, or attitudes are only exhibited at the request of a teacher or tester, they are unlikely to appear organically in another situation. In classroom settings, habits of mind are observable when students are given opportunity to make independent decisions about their work processes and products.

There are eight Studio Habits of Mind: Develop Craft, Engage and Persist, Envision, Express, Observe, Reflect, Stretch and Explore, and Understand Art Worlds. When students *Develop Craft*, they learn techniques, artistic knowledge, and proper tool usage. This Studio Habit also includes setting up one’s workspace, caring for materials, and cleaning the studio to be shared by all. *Engage and Persist* can be seen when teachers make sure to allow student interest to play a part in the class, and actively help students recognize what engages them. When students are authentically engaged, persistence through challenges that arise in the artmaking process happens naturally. *Envision* is a synonym for imagine – in art, students use their imagination to create a plan, a vision for their work, to manage their time and predict how long processes will take, and see various possibilities for making changes to their work. Art teachers encourage use of subject matter and media choices, as well the artistic elements and principles to help students *Express* meaning and feeling in their creations. When making art, teachers and students also *Observe* closely – they don’t superficially glance at their or others’ artworks or at their environment – they notice and look with sensitivity. *Reflect* most often happens in one of two forms – one is *Evaluate*, in which students comment on their own and others’ artworks in terms of what pleases them and what bothers them; the second is *Question and Explain*, which is how teachers encourage metacognition, as students talk about their process, what worked, what didn’t, and how they were inspired to make the artwork. Teachers encourage students to *Stretch & Explore* by allowing time for play, discovery, and “mucking around” – sometimes through center-based activities, media explorations, or simply by encouraging a student to go forward with a risky decision about modifying an artwork. The final Studio Habit, *Understand Art Worlds*, is seen when teachers help students to recognize that what they are working on in school connects to what professional artists work on, and to recognize that there is an art world out there in which collaborations of artists, curators, art historians, media, and critics have together shaped the rules and guidelines and canon of the visual art domain.

The Studio Habits of Mind emerged from naturalistic observation of authentic processes of creative making in the classroom. While widely used by arts educators (Hogan et al., 2018), this use of this framework has never been empirically investigated in professional artists. We chose to study this by analyzing the behavior and talk of fledgling fashion designers on the television show, *Project Runway.* This allowed us to capture artists in a naturalistic, creative work environment. This footage was ideal because contestants are constantly required to speak with producers in “confessionals” on camera, and to interact verbally with other contestants, their mentor, and the show’s judges. Given our focus on artistic process, influenced from art education, which depends on listening to creators reflect on their work, the reality show setting allowed us to look at patterns of thinking.

The aim of the study reported here is to demonstrate how the Studio Thinking framework can be used as a way to illuminate habits of mind, or thinking dispositions, during creative acts. Unlike any currently accepted approaches, the process identified here has applicability to other domains to help researchers examine what it means to be creative though a lens that is not dependent upon numbers, ranking, or other quantitative paradigms.

## Materials and Methods

### Dataset

*Project Runway* is an American reality television show that premiered in 2004. The show serves as both a platform to showcase talented up-and-coming fashion designers and as a way to illuminate the intricacies of the design process for viewers. In the words of Heidi Klum, renowned supermodel and the show’s host, “we knew that designing is a really creative, interesting, inspiring process, and that it wouldn’t be a boring hour of watching people sew” (Mell, 2012).

The show has run for 16 seasons (186 episodes), and six spin-offs have been created, including *Project Runway: All Stars* for returning designers and *Project Runway: Junio*r for teen designers. Additionally, 28 international versions exist including *Project Runway Middle East, Mission Catwalk* (Jamaica), *Project Runway Philippines*, and *Project Catwalk* (Netherlands*;* “Project Runway,” n.d.). The show’s popularity has resulted in 81 Emmy nominations and six wins, including a nomination for Outstanding Reality-Competition Program every year since 2005. The show is immensely popular and reaches viewers not only in the United States, but around the world.

In each episode, designer-contestants compete against one another to create garments for the given challenge of the week. One of the lowest scoring designers is eliminated each week, as determined by three permanent judges from the fashion industry (Klum, fashion magazine *Elle*’s editor in chief, Nina Garcia, and American fashion designers Michael Kors [seasons 1–10] and Zac Posen [seasons 11–16]) and one rotating guest celebrity judge. The last remaining three (or sometimes four) designer-contestants are given time and financial resources to design a complete collection to be premiered at Fashion Week in New York City. One final season winner is chosen from these finalists.

Each episode follows a prescribed format: a preparation period (contestants are first assigned a challenge and given time to prepare, sketch, and shop), worktime (contestants spend time constructing in the workroom, seeking feedback from fellow-contestants and mentor and show co-host Tim Gunn), and finally the runway (a presentation and judging of garments on the runway.) Each episode features a unique challenge. Sometimes contestants must collaborate in groups. Other times, challenges constrain the designers, for example to avoid textiles and instead use materials from unexpected locations, such as a flower shop (Season 2), a candy store (Season 4), or a pet store (Season 9; Heching, 2017). *Project Runway* challenges have included avant-garde fashion, toddler wear, dog clothes, outfits for stiltwalkers, professional wrestling outfits, drag costumes, and “everyday woman” challenges which include average people of all shapes and sizes as models.

### Coding Manual

We selected Seasons 8 and 9 of *Project Runway* for the development of a coding method and coding manual. These seasons were chosen because they fall at the mid-point of the show’s 16 season run. All 28 episodes were transcribed and verbal statements by all persons on the show were coded using the Studio Habits of Mind framework. Four researchers coded these two seasons using the online coding platform Dedoose.

During coding, a deductive process was used (Crabtree and Miller, 1999), with eight codes reflecting the Studio Habits of Mind (develop craft, express, envision, engage and persist, observe, stretch and explore, reflect, and understand art worlds; Hetland et al., 2007, 2013). Our manual included example behaviors and statements sorted into the appropriate Studio Habit of Mind. The manual included three levels of information: the code label (the Studio Habit of Mind), what the code concerns (a sub-grouping/short definition, based primarily on Hetland et al., 2007, 2013), and a description of what the code sounds like within the context of a Project Runway episode (including guidelines for using or not using the code; Boyatzis, 1998; MacQueen et al., 2008). Coding was an iterative process: the P.I. and three coders independently coded transcripts and returned to the group to discuss decisions and the fundamental characteristics of each Studio Habit of Mind as outlined in *Studio Thinking 2: The Real Benefits of Visual Art Education*. This process underwent several rounds of individual coding, followed by group meetings to compare observed behaviors to definitions from Hetland et al. (2007, 2013). While exemplars of behaviors differed between those identified in Hetland et al. (2007, 2013) and what was observed on Project Runway, all examples retained the fundamental definitions of each Studio Habit of Mind as defined by Hetland et al. (2007, 2013). Researchers engaged in a process of constant comparison (Glaser, 1965) throughout Seasons 8 and 9 in order to make sure various manifestations of each code were included in the example section of the manual. This process also included periodic checks for inter-rater reliability across coders, discussion of discrepancies, and clarifications to the manual. Additionally, those researchers creating the coding manual engaged in periodic peer debriefing (Lincoln and Guba, 1985) with the fifth research team member.

### Data Coding

All 14 episodes of Season 10 of Project Runway were selected for analysis using the coding manual developed with Seasons 8 and 9. Episodes each averaged 63 min of content. Three research team members participated in coding of Season 10. These were also transcribed and coded in the online coding platform Dedoose. Nine of the 14 episodes were coded individually by one of three coders (each coder independently coded three episodes). Three were coded by two independent coders (each person in the pair coded separately in order to calculate inter-rater reliability). The pooled Cohen’s kappa of these episodes averaged 0.84 which is considered good to excellent agreement (Fleiss, 1971; Cicchetti, 1994; Miles and Huberman, 1994). The last two episodes were coded consensually by the three-person data coding team (these are finale episodes that include an unusual format – visits to the designer-contestants’ homes by Tim Gunn and the preparation for and presentation at New York Fashion Week). The decision to code these two episodes consensually was made prior to beginning data coding.

The show’s structure switches frequently between two formats: the primary action of the show and confessional-style reflective interviews with individual contestants. With each switch, a new unit of analysis began. Each particular code could be assigned only once per unit of analysis, but unlimited types of codes could be assigned per unit of analysis. Some units of analysis received no codes because no Studio Habits of Mind were exhibited. During portions of the show that were on the runway, this scheme created units significantly longer than the other two sections. Therefore, for this part of the show, we switched units when a new judge began critiquing a designer. When the designers left the runway, we switched units when the judges began a conversation about a new designer.

## Results

To reiterate, our goal was to answer the following two questions: *How do creative people act and think while engaged in creative behavior*? and *Can we systematically capture the thinking dispositions of creative people as we observe them at work?* These questions are not answerable by current approaches to creativity research. We used the Studio Thinking framework, shown to be useful in visual art education, as the framework for systematizing collected data.

The most important finding is that we saw abundant instances of each of the eight Studio Habits in the *Project Runway* episodes. These did not stray from the original definitions and descriptions as put forth in *Studio Thinking* (Hetland et al., 2007, 2013), but examples specific to this fashion design setting do of course differ from those seen in the high school classroom art studio (the ways in which this happened were uncovered and notated within the creation of the coding manual). This translation of the framework to another setting shows that the framework can be used as a lens for looking at creative and artistic behavior outside of the art studio-classroom. In this section, we describe examples of each Studio Habit of Mind displayed on *Project Runway*, in alphabetical order.

### Studio Habits of Mind in Fashion Design

Because the habits work in conjunction with one another (Hogan et al., 2018, p. 44), examples described below may demonstrate more than one Studio Habit of Mind. During the coding process, all appropriate codes would have been applied.

#### Develop Craft

Designers and judges regularly discussed technical abilities of garment construction, and the effect these had on other Studio Habits–like the impact construction mistakes had on being able to express the appropriate feel of the garment, or a mistake being very obvious to an observer. These are the skills of being a fashion designer – choosing fabric, budgeting, constructing and fitting a garment, styling and editing, adding make-up and hair style, and presenting on the runway. Codes for Develop Craft often reflect how designers use these technical skills to make other informed decisions about what their garment will look like, or how they will change it. Without technical skills, a creative vision cannot be achieved. Develop Craft was seen during judging, as shown in this critique of technical skill and styling from judge Michael Kors in Episode 3: “The skirt was a piece of fabric. It literally, just gathered at the waist. Crooked hem, with that ugly red belt in the wrong place.” Develop Craft was also seen in this critique of fabric selection and compliment of silhouette design in Episode 7:

I think that when we look at, you know, [the garment of designers] Gunnar and Kooan, it could have been a really fabulous gown, but I think they picked the wrong fabric. But do I think it’s a great silhouette? Do I think the back of it was really pretty? I like the chiffon. She looked gorgeous. The silver at the neck was fabulous. But I think there were some fabric issues.

In addition to discussion of technical skills during judging, during their worktime the designers discussed the importance of technical skills and the consequence of not having them, as in this excerpt from Episode 10:

[Designer] Melissa: Fabio, my zipper fell off!
[Designer] Fabio: Hold on. Don’t—hold on to it. Did you sew the top of it?
Melissa: No, I forgot. This is not good.

#### Engage and Persist

Designers showed signs of Engage and Persist when they found personal engagement in the work process, became immersed in garment making, buckled down to find solutions to problems, and made compromises for the sake of time management. The most simplistic form of Engage and Persist was when designers displayed satisfaction and focus in their work. In Episode 10, designer Sonjia declares, “I love making over-the-top kind of pieces, so for me, this—this challenge is exciting,” showing her engagement in the work process. On the other hand, in Episode 14, designer Christopher explains his lack of engagement, which affects his work process, “It’s so emotional and physically draining. It’s just too much to deal with at once.”

This code was also used for instances in which designers specifically mentioned their inspirations, or sources of engagement. For instance, as designer Dmitri introduces his collection at New York Fashion Week in Episode 14, he says, “My inspiration for this collection was organic architecture. I’m proud of what I did. I hope you guys like it.” Other times, this Studio Habit of Mind appears when problems need to be solved, and focus was required, which was often due to the time constraints (most garments must be completed in one day). This is exemplified in mentor and co-host Tim Gunn’s signature phrase, “Make it work!”, which refers to making the best of situations, and persisting to complete one’s look, even if not to the standard of the designer’s original goals. As he tells the designers before departing the work room in Episode 14, “This is about making it work. If there ever were a make it work moment, it is this one. Off we go!” Other times, this is a message specific to design issues, as in this Episode 7 moment as the group departs the work room and heads to the runway:

Tim: Sonjia, why are you freaking out?
Sonjia: I ran out of time, and it’s just–
Tim: She looks good.
Sonjia: The hem’s not done, and I didn’t put enough room for the zippers. She couldn’t get into it, and then I had to hand-sew the zipper, and it’s just not what I would do, like I-
Tim: That’s all right. As long as she can– as long as you fake it on the runway, it’s gonna be fine, okay?
Sonjia: Thank you.
Tim: Remember, channel your inner winner, okay?

#### Envision

Instances in which designers used their imaginations were coded with Envision. These included considering ideas for one’s work, or making a plan for reaching those imagined visions. In Episode 10, designer Ven discusses what he imagined for his model’s eyeshadow with the makeup designer.

[Designer] Ven: So, this is the fabric [shows the pattern of dress fabric], and I really want the focus to be the eyes.

Make up artist: Start with a highlight, right in the center.

Ven: And then fade it out to a color. Oh, that’s perfect.

Ven’s conversation shows how the designers often have very precise visions for their work. When working with other designers, hairstylists, or makeup artists, they try to articulate this vision and know whether or not it’s been achieved. As Ven comments in Episode 6, “[My model] Terri comes in for the fitting and her hair looks beautiful. It’s exactly the direction that I was going for.”

These visions also affect the plans that designers must make in order to achieve them. In Episode 11, designer Melissa has to rethink her plans as the challenge includes a last-minute “twist” in which designers must create garments for not only for a child, but also a complementary adult outfit. “I really have to change my course of action. I am going to…cut the white denim into a dress, and do a drape kind of shift dress for the little girl.”

#### Express

Designers regularly used their garments to convey a meaning, feeling, or message. They also used them to express their own personality, style, and individual signature as a designer. This is often articulated in the detailed descriptions of the woman they are theoretically designing for. Sonjia speaks about her muse in Episode 4:

I wanted to create a look for a woman who has a lot going on during the day so she’s probably running errands in the morning, in the office during the day and basically something that can take her from wearing her hair up to down to, you know, flats to pumps to basically anything she wants to wear.
Conveying associated moods and feelings are not only part of the designer’s process, but also part of the experience of viewing a garment, as often articulated by the judges. In Episode 4, the judges respond to the work of Fabio and Ven.
Michael Kors: The mohair coat’s a full flop.
Fabio: Oh.
Michael: I mean, to me, it’s a Grandma housecoat. She should have Kleenex in her pocket. I mean, it’s just—
Heidi: It just hangs.
Michael: It’s sad. What I’m mystified is, where are you in all of this? None of this looks like anything that you would ever touch.

Michael’s comment, “where are you in all of this?” refers to the signature styles each designer expresses through their work – so much so, that when something is out of character, like the grandmotherly feel of Fabio’s jacket – it is notable.

#### Observe

When attention was called to something that wouldn’t ordinarily be seen, Observe was coded. This sometimes was an observation that came from close inspection (like comparing a garment to something else), or from a critique of something that required careful looking to see. When someone asked to see a garment in a different way (from the back or side, with a jacket removed), this also revealed careful looking and was coded as such. These types of codes appear in this excerpt from a judging session in Episode 4:

Michael Kors: It looks like a hairdressing smock. Like she was cutting her hair, she—you know, there was a fire in the beauty salon, she belted it, and she ran out in her zebra dress, and the whole thing is just weird.
[Guest Judge] Hayden Panettiere: Can you lift up the coral [part of the dress]?
Designer Buffi: Yeah.
Michael: Well, the hem is cuckoo, too.

#### Reflect

This is the only Studio Habit of Mind which we treated specially due to the fact that our context was reality television footage. Because of the nature of the program, all cast members were constantly put in situations in which they were asked to recall for the camera what had just happened, or the steps of their work process. Therefore, Question and Explain (one portion of Reflect) happened frequently, but artificially due to the nature of the reality television situation. For this reason, we limited Reflect codes to those of the other Reflect sub-habit, Evaluate.

Reflect codes were given for any assessment or critical analysis of one’s own or another’s work. These occurred in all possible pairings of cast members – designers evaluated each other’s work and work process, Tim Gunn and the judges evaluated designer work, and even designers evaluated the judging competency of the judges. In Episode 7, Fabio reflects positively on Dmitry’s design, “I like Dmitry’s dress because the fit, that is, like, so form-fitting, but at the same time, so effortless” while designer Christopher evaluates the datedness of Sonjia’s work negatively, “Sonjia, the 80’s called and they want everything back. Cyndi Lauper is missing a dress and a clutch.”

Sometimes reflections were given more generally about a designer’s relative strengths and weaknesses, or about his or her broad trends in working. In Episode 7, designer Alicia is both complimentary and critical of Christopher’s technique use: “Chris does a lot of the same stuff. He does a flowy gown; he does this textile thing, raw-edged silk, and it’s cool, but when you keep doing it over and over again, I don’t want to see it anymore.”

#### Stretch and Explore

This code was most commonly given when taking risks or breaking out of one’s comfort zone was discussed. For instance, in Episode 7, Christopher addresses the critique from Alicia above about using the same technique multiple times, “Yes, I’ve done this technique for the first challenge, and for the skirt in the *Marie Claire* challenge. It’s kind of getting, you know, old, and it’s a huge risk that I’m taking.” In this statement, he acknowledges that trying new things is part of what is expected of him as a fashion designer. These are expectations that the designers have internalized – Fabio says in Episode 13, “I just hope that [the judges see] that I am pushing myself as a designer, but I’m also pushing the boundaries on design.” The judges and mentors were often coded for encouraging these types of behaviors. In Episode 2, Tim Gunn reiterates this to the designers as he leaves them to work, “I just want to encourage everybody to really push at the boundaries. Wow the judges.” While discussing Fabio’s work in the avant-garde challenge of Episode 12, Michael Kors jokes, “Out of all of our designers, you don’t have to ask him to be avant-garde. He’s playing with proportion. He’s playing with gender roles. I mean, this guy is thinking outside the box.”

#### Understand Art Worlds

Working with others and having an understanding of the larger domain in which one is working are the two primary tenets of Understand Art Worlds, and both were present in *Project Runway*. This code was given when designers talked about both positive and negative aspects of the unavoidable collaborative process in the real world of fashion design and clothing production. In Episode 3, designer Elena talks about the challenges of working with someone not as skilled: “I’m realizing now that [my partner] is not going to be able to help me with the construction of the dress. She’s moving at a snail’s speed. I’m handling this by working even faster.” Melissa reflects on the help she receives when her zipper unexpectedly breaks in episode 10: “So, Fabio tries helping me; Sonja tries helping me; Christopher tries helping me get this little freaking zipper back on. It’s not happening.”

Understand Art Worlds also encompasses the additional understandings needed as a member of the fashion community. As Elena says in Episode 4, “The fashion industry is a shark. If you can’t handle it, then maybe you shouldn’t be in this industry, because that’s the way it is.” In coding, this included concepts like whether garments are sellable, whether they are constructed properly for their purpose (like toddler-proof child clothing, or bold designs that can be seen from afar on a pop star’s stage costume), and the referencing of famous fashion designers’ previous designs. These codes often appeared in challenges that included prizes that brought designs out to the community – like the department store Lord and Taylor challenge, for which the prize was a contract to have the created garment reproduced and sold in stores. Judge Heidi Klum critiques designer Elena on her garment in Episode 7:

You have to think that you want to sell. I think that this is a very sellable dress. I think that a lot of women are attracted to this kind of silhouette…I think it’s a very flirty and fun kind of a dress.

Later in the episode, the judges discuss Melissa’s knowledge of marketability:

Heidi Klum: Melissa did a good job today, you know, which is nice. She’s really cool and edgy. It was nice to see something different.
[Guest Judge and Lord and Taylor representative] Bonnie Brooks: I think it would look great in the window.
Michael Kors: Hers is the most dramatic.
Nina Garcia: It felt very modern. It was dramatic. Yet it’s wearable.
Michael: Listen, this is the most dramatic–Melissa’s– but it’s the toughest, probably, of our favorites to sell.
Bonnie: I think so.

#### Habit Frequencies

We have shown here ample examples of each of the Studio Habits of Mind in the behavior and talk of fledgling fashion designers. In Table 2, we include tallies of each Studio Habit of Mind to show how prolifically each was included in our analysis. Because reality television shows undergo considerable editing, we avoid claims about the proportions of certain Studio Habits or in which sections particular habits appear. We include these numbers simply to show that the instances of codable Studio Habits of Mind talk and behavior were not in any way rare.

**Table 2:** Studio Habits of Mind frequencies.

	Averagelength	Developcraft	Engage and Persist	Envision	Express	Observe	Reflect	Stretchand Explore	UnderstandArt Worlds
Total	63 min.	410	747	343	354	283	1137	74	903
Preparation	12 min.	50	134	80	51	9	63	5	190
Worktime	23 min.	149	335	181	111	102	400	24	300
Runway	27 min.	165	156	47	147	151	543	34	253


*Preparation, Worktime, and Runway figures reflect episodes 1–12 only; Total figures reflect all 14 episodes of season 10.*

## Discussion

We have proposed two not commonly used methods for the study of creativity: examining the broad thinking dispositions, or habits of mind, that govern the act of artistic creation (instead of quantitative, product-based measures), and using footage from a reality television show as a source of data.

The Studio Habits of Mind (Hetland et al., 2007, 2013) are widely used in primary and secondary school visual art education. Administrators use them to identify quality arts education, teachers use them to assess their students’ thinking, and students use them as a way to practice metacognition during artmaking (Hogan et al., 2018). We argue here that a habits of mind framework can be used to investigate creative behavior in a variety of settings, and the applicability of the Studio Habits of Mind to the design process illustrated on *Project Runway* is an example of how this can happen.

Through deductive qualitative analysis, we have answered our research questions: *How do creative people act and think while engaged in creative behavior*? and *Can we systematically capture the thinking dispositions of creative people as we observe them at work?* In these examples of fashion designers, we find ample evidence of all Studio Habits of Mind during the work process. The Studio Habits of Mind provide a systematic lens for capturing the thinking behaviors (as evidenced through the spoken words of fashion designers) during the act of garment design. We view this as initial evidence of the validity of this framework for looking at creative behavior, and hope it serves as a catalyst for other creativity researchers to think more deeply about the examination of creators as they work.

### Purposes of Assessment Tools

It is important to note that assessments of creativity needn’t always be high-stakes, and we do not suggest that the approach articulated here be used alone in high-stakes assessment situations. Some situations require ranking, cut-off scores, or other means of quantitative sorting. But many do not. The approach described here provides an alternate lens for looking at creative behavior – one already shown to be useful for teachers who think about the work processes of their students, and one which could be adopted by creativity researchers as a way to illuminate other parts of the creative process not captured by current measures. For instance, Engage and Persist is not a habit of mind we see encapsulated within current approaches (though these constructs may appear in personality measures, they do not exist in measures within the context of creative behavior), yet anecdotal and historical evidence of highly creative people shows that many creators are extremely persistent and deeply engaged in their processes (Gardner, 1993). Without looking systematically at the behaviors of those who participate in creative acts, how can we know which aspects of creative behavior to choose to measure quantitatively?

Qualitative investigations can help researchers as they develop new objective and quantitative measures more suitable for traditional psychological means. For instance, Hogan et al. (unpublished) have created quantitative measures of some of the Studio Habits of Mind for primary school aged students. And an international research project by the OECD in assessing creative habits of mind (Lucas and Spencer, 2017) has led to the development of a creativity section on the PISA (the international assessment used to compare educational systems) to be administered in 2021 (Lucas, 2017). In both of these examples, qualitative, habit of mind-based approaches have helped to inform and inspire the creation of new quantitative measures.

We believe the adoption of new approaches is particularly important as the ways in which we look at creative behavior continue to expand. For many years, investigations of creativity were grouped into one of two groups: Big C (eminent, domain-changing creativity) and little c (every day acts of creativity). But as Kaufman and Beghetto (2009) suggest, our understandings of creativity can be broadened to include not just famous Big-C creators like Einstein or Picasso, but also categories like Pro-C (professional expertise, like that found in the average office or in the workroom of *Project Runway*) and mini-C (transformative learning, as is found in art classrooms like those in which the Studio Thinking framework was developed). As our classifications of “creativity” continue to expand, the ways in which we examine these behaviors should, as well.

### Our Approach and the 4 Ps

We don’t see any of the current approaches described earlier in the paper as ones that can answer our research questions: *how do creative people act and think while engaged in creative behavior*? and *can we systematically capture the thinking dispositions of creative people as we observe them at work?* We do, however, see similarities and differences between our approach and those of some of the 4 Ps. Distinctions of “process” and “product” are blurred when using a disciplinary thinking or habit of mind approach. So while the spirit of looking at the procedures that lead to creative artifacts (or products) is shared between our visual arts education-influenced approach to process and process approaches of psychology, these differ in their qualitative and quantitative approaches.

Our goal to create an ecologically valid, discipline and situation dependent approach shares similarities with ideas put forth by Amabile. The product-based approach of the Consensual Assessment Technique (Amabile, 1982) acknowledges the contextual distinctions of what can be considered creative. Perhaps most similarly, environment (or press) approaches often use frameworks for looking at characteristics of workplaces (Amabile often looks at indicators of sources of motivation by workers, e.g., Hennessey and Amabile, 1988; Amabile et al., 1996; Amabile, 1997), and how those may influence creative behavior. We see our subjective approach as similar, but rather than looking at environment, we focus on evidence of thinking by the creator.

It is possible that some creators would report personality characteristics related to some of the Studio Habits of Mind – like persistence (Engage and Persist), free-thinking (Stretch and Explore), or imagination (Envision). But rather than rely on self-report of general personality characteristics, we think a third-party observer of these thinking dispositions *during* the act of creating is more useful and potentially more reliable.

### Limitations

There are several considerations that future researchers should review when applying similar methodologies.

#### The Relationship Between Artmaking and Creativity

It is important to note that the Studio Habits of Mind emerged in the process of studying artmaking, without specific regard for creativity. Artmaking is not always creative (as in paint-by-number activities, or step-by-step art class activities sometimes used by art educators), and creativity can be found in many domains besides artmaking.

However, the Studio Habits of Mind are related to what is required in creative behaviors, and there is a natural connection between art and creativity. As Hetland and Winner (2011) point out, creative and artistic thinking dispositions share several qualities: they tie subjects together interpretively (Perkins, 1994; Efland, 2004), allow for adaptive novelty (Perkins, 1981), and are situations in which an individual interacts with a field and a domain (Csikszentmihalyi and Csikszentmihalyi, 1992). Additionally, even a superficial glance at the Studio Habits of Mind suggests connections to lay understandings of creativity. Stretch and Explore includes taking risks and learning from mistakes; committing to solving a problem is exemplified by Engage and Persist; Understand Art Worlds and Observe call for a critical awareness of what’s going on around you.

More systematically, we can map aspects of the Studio Habits of Mind onto more formalized definitions of creativity. Consider Guilford’s (1967) view of convergent and divergent thinking. Thinking divergently requires a willingness to Stretch and Explore and Envision new possibilities, while convergent thinking requires Understanding Art Worlds (to understand conventions), Develop Craft (to be able to execute those conventions), and Observe (to have an awareness of what’s going on around you.) While not precisely the same, we see a clear resemblance between strong artistic thinking and creative thinking.

#### The Nature of Reality Television and Bias

Reality television footage is not untouched reality – it has gone through many hands in an editing process. The editors of reality shows have considerations beyond showing an authentic work process: they must create enough “drama” to maintain viewership and to properly include reference to sponsoring products or organizations. Each reality show has its own aims, and not all are appropriate as a source of data. However, we believe that in many cases of reality television, the viewer is a witness to the creative process.

*Project Runway* is particularly notable for minimizing “drama” and keeping the work process at the center. In fact, the show was praised for the authentic way that it uses the television reality contest genre to “engage, enlighten, and inform,” when given a Peabody Award in 2007 (Project Runway, n.d.). Hendershot (2009) also notes this in her analysis:

This is not a series driven first and foremost by character conflict. [*Project*] *Runway* producers choose to show long sewing sequences in the Parsons School of Design workrooms rather than focusing on personality issues back at the apartments that the designers share. In fact, contestants are only occasionally pictured there…Here, if people’s issues do come up, it is only a distraction from the work that must be done.

Producers also emphasize that the creative process is at the heart of the show. After noticing that full open calls to find designers meant “too many people were coming in who were clearly less interested in design than they were interested in being on TV,” (Mell, 2012), they cut back to only one or 2 days of open calls in New York and Los Angeles, and now use casting directors across the country to find twenty to thirty contestants for the casting judge panel. When asked if she thought the designers are their true authentic selves on the show, Desiree Gruber, one of the show’s producers, responded:

I wouldn’t believe if somebody said they were able to hide their true personality throughout the whole season. It’s too stressful. I think one of the reasons the show is so popular is that viewers get into the act of creating along with the designers. We’re following people who are authentically very creative; it’s not manufactured. They’re trying to bring out their best, which is hard to do in a timed experience. Being creative under pressure is not easy (Mell, 2012).

Even in instances when former designer-contestants have complained to popular media outlets about possible injustices regarding predetermined winners or unfavorable editing, they admit that the challenges and work process are very real (Wayne Hughes, 2012; Forbes, 2015; Berman, 2017). In short, while many factors go into the editing of reality television, we feel confident that for the purposes of looking at the creative process over time, footage from *Project Runway* is a useful and valid dataset.

Footage from many kinds of reality shows can provide both researchers and the general public an easily available data source for understanding the creative making process. The popularity of shows like *Project Runway* makes analysis and results accessible to a broader audience – both among researchers and with the general public. In addition, it is a way for researchers to look at creative behavior without the particular limitations of an artificial laboratory setting. Finally, this source of data is widely available and easily accessible.

We acknowledge potential issues of bias as a result of editing for television. However, we believe this issue is minimized because of the research question and the coding methodology applied here. Were this a grounded theory study, in which data was inductively analyzed for the emergence of habits of mind (such as that reported in Hetland et al., 2007, 2013), edited footage would be problematic. We would not know what habits of mind were contained in those parts not deemed worthy of television footage. But since we used a deductive approach, mapping a pre-existing framework on to footage, and since we found evidence of all Studio Habits, we argue that this dataset is useful for the purpose of answering the research questions put forth in this study.

### Broader Impact and Future Directions

The ideas put forth here can be useful to two primary groups: teachers and researchers.

#### Teachers

Many art teachers already regularly use the Studio Habits of Mind in their classroom language, curriculum planning, and assessments. Videos of contemporary artists at work can help illustrate these concretely for students (such as videos on Art21; see Hogan et al., 2018). Excerpts from some reality shows, including *Project Runway*, can also be used to exemplify the Studio Habits of Mind at work in a way that is engaging and relevant to students. While these excerpts should be carefully chosen and screened for school appropriate themes and language, much of what we viewed in our coding procedure could be shown to students (particularly high school students) to help illustrate Studio Habits of Mind and foster class discussion. Educators in other disciplines have used reality television in the classroom (such as connecting social studies to *The Amazing Race* [Weddell, 2011], using *Undercover Boss* and *Bar Rescue* in management classes [Quain et al., 2018] and using reality shows as a model for designing classroom activities [Bach, 2011] or as models of good and bad teacher behavior for critiques [Higdon, 2008]).

#### Researchers

People naturally associate artistic endeavors, like fashion design, with creativity. But of course much of creative behavior happens in realms outside of the arts. The Studio Habits of Mind are broad and have potential to be relevant to all domains. This research method need not be limited to a lens for looking at artistic endeavors but can be expanded to look at creativity in domains not traditionally associated with creativity, including cooking (as in Food Network’s *Chopped*), tattoo design (exemplified by Spike TV’s *Ink Master*), hair design (like Bravo’s *Shear Genius*), or even dog grooming (seen on Animal Planet’s *Groomer Has It*). Of course, while reality television competition shows are convenience samples, a researcher can also use unedited filmed data or in-person observations.

We propose that the Studio Habits of Mind be used, at minimum, as an initial framework for systematic qualitative analysis of creative behaviors. Use of the framework, while commonly accepted in arts education, should be replicated for its utility and applicability in other places. We have begun this here with a look at the domain of fashion design. More work is needed to see whether and if so how these habits are used in other professional artistic areas, and/or outside of traditional arts disciplines.

It is possible that for some domains, additional or different habits of mind are more relevant to creative behaviors. In situations in which the Studio Habits of Mind seem inauthentic, we encourage researchers to use a two-part study to examine what habits of mind emerge most frequently in different domains by using the grounded theory approach of Hetland et al. (2007, 2013) and then replicate and examine those habits of mind for validity in different settings, as we have done here.

## Author Contributions

JH conceived of the study. MH and KM contributed to literature reviews. JH, MH, KM, and AL designed the coding manual and coded the data. JH analyzed the data and wrote the paper with input from KM and EW. EW supervised the study.

## Conflict of Interest Statement

The authors declare that the research was conducted in the absence of any commercial or financial relationships that could be construed as a potential conflict of interest.
